# Supplementary material for: NAc-VTA circuit underlies emotional stress-induced anxiety-like behavior in the three-chamber vicarious social defeat stress mouse model
Source: Nat Commun. 2022 Jan 31;13:577. doi: 10.1038/s41467-022-28190-2 (PMC8804001; doi:10.1038/s41467-022-28190-2)
Supplement: Supplementary file 1 — Supplementary information [file 41467_2022_28190_MOESM1_ESM.pdf]

SUPPLEMENTARY INFORMATION

NAc-VTA circuit underlies emotional stress-induced anxiety-like behavior in the three-chamber vicarious social defeat stress mouse model

Qi et al.

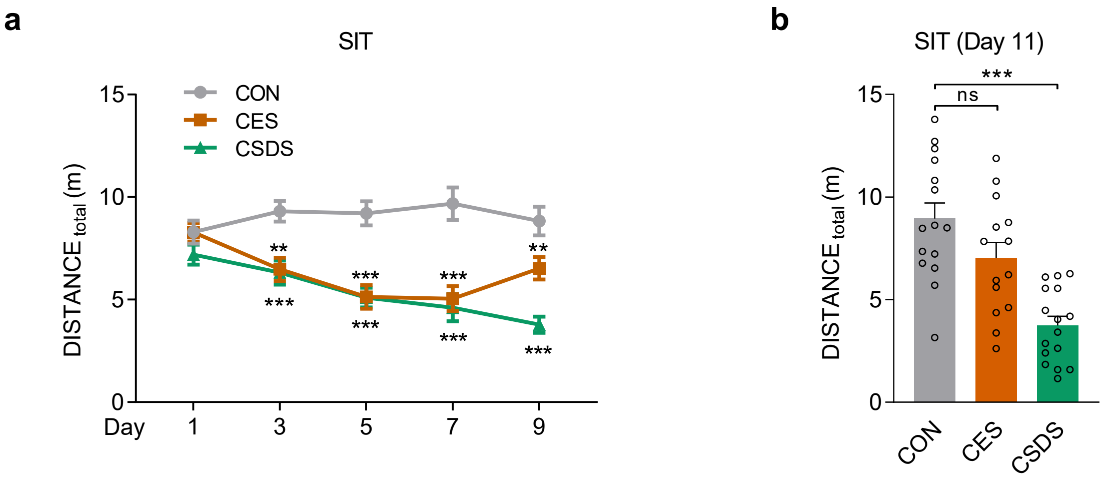

Supplementary Figure 1. The total distance travelled in the SIT arena.

(a) Distance travelled in the SIT arena at different time points (two-way RM ANOVA, group: $P = 6.29 \times 10^{-12}$, time: $P = 0.0014$, interaction: $P = 0.0011$; post hoc Tukey's test, day 1: CON vs. CES: $P = 0.9999$, CON vs. CSDS: $P = 0.3649$, day 3: CON vs. CES: $P = 0.0025$, CON vs. CSDS: $P = 0.0009$, day 5: CON vs. CES: $P = 7.05 \times 10^{-6}$, CON vs. CSDS: $P = 2.77 \times 10^{-6}$, day 7: CON vs. CES: $P = 2.96 \times 10^{-7}$, CON vs. CSDS: $= 6.85 \times 10^{-9}$, day 9: CON vs. CES: $P = 0.0047$, CON vs. CSDS: $P = 7.72 \times 10^{-9}$). **(b)** Statistical results of total distance travelled on day 11 (one-way ANOVA, $P = 5.30 \times 10^{-6}$; post hoc Tukey's test, CON vs. CES: $P = 0.1219$, CON vs. CSDS: $P = 3.64 \times 10^{-6}$). n = 15 for CON mice, n = 14 for CES mice, and n = 16 for CSDS mice. Data are shown as mean ± SEM. Compared with control mice, $^{*}P \leq 0.05$, $^{**}P \leq 0.01$, $^{***}P \leq 0.001$, ns, not significant.

a

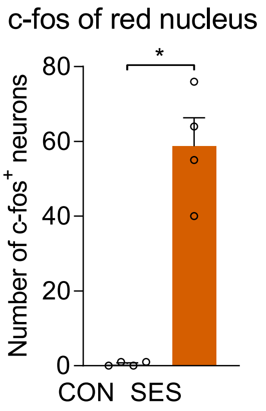

Supplementary Figure 2. Qualification of c-fos-positive cells in red nucleus.

(a) Statistical results of c-fos-positive cells in CON and SES mice (two-tailed Mann Whitney test, *P* = 0.0286). n = 4 mice for each group. Data are shown as mean ± SEM. Compared with control mice, *$P \leq 0.05$.

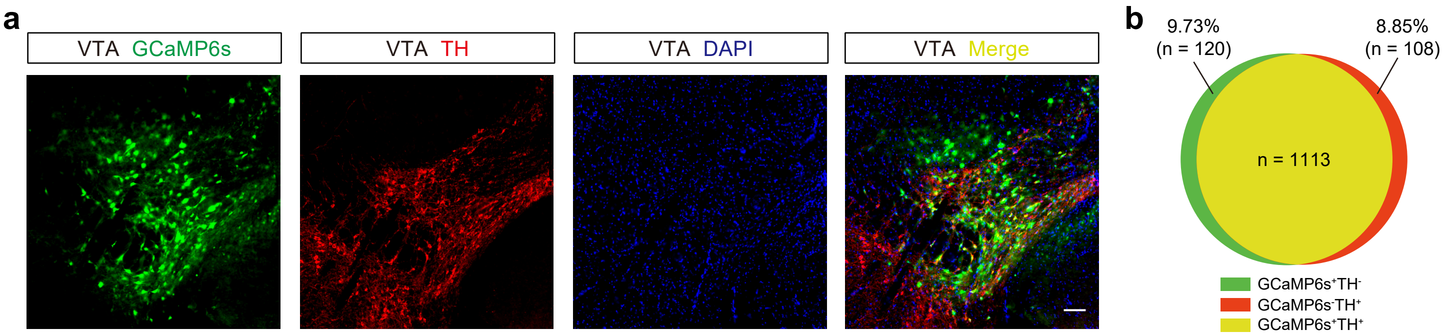

Supplementary Figure 3. Representative photographs and quantification show double staining of TH and GCaMP6s in the VTA (to Fig. 2i).

(a) Representative images show the co-expression of GCaMP6s with TH in VTA (green, GCaMP6s; red, TH; blue, DAPI). Scale bars, 50 μm. **(b)** The quantitative analysis of Venn diagram shows the co-expression level of GCaMP6s with TH in VTA (3 sections per mouse from 5 mice).

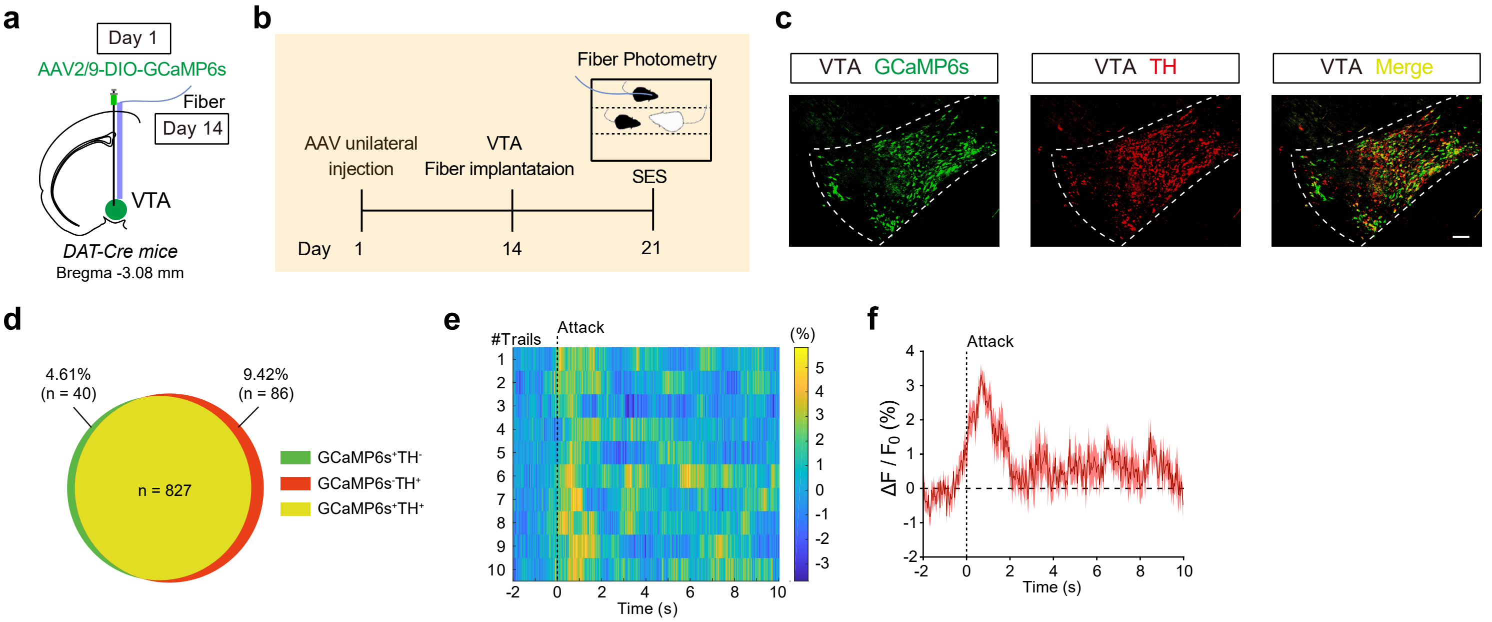

Supplementary Figure 4. Ca^{2+} signal of VTA^{DA} neurons of DAT-Cre mice increases in anxiety-inducing events.
(a) Schematic of GCaMP6s infection and optical fiber implantation in DAT-Cre mice. **(b)** Experimental scheme of anxiety-inducing events. **(c)** Representative image of VTA injection sites. Scale bar, 100 μm. **(d)** The quantitative analysis of Venn diagram shows the co-expression level of GCaMP6s with TH in VTA (3 sections per mouse from 3 mice). **(e)** Trial-by-trial heatmap of anxiety-inducing events-evoked Ca^{2+} transients. The Black dotted line represents the onset of anxiety-inducing events exposure. Each trail represents the target mice observed their conspecific received attacks by CD1 aggressor. **(f)** Peri-event plot of average Ca^{2+} transients (n = 3 mice). Black dotted line, the onset of emotional stress exposure (target mice observe a conspecific attacked by CD1 mice). Surrounding shaded areas indicate error bars (SEM).

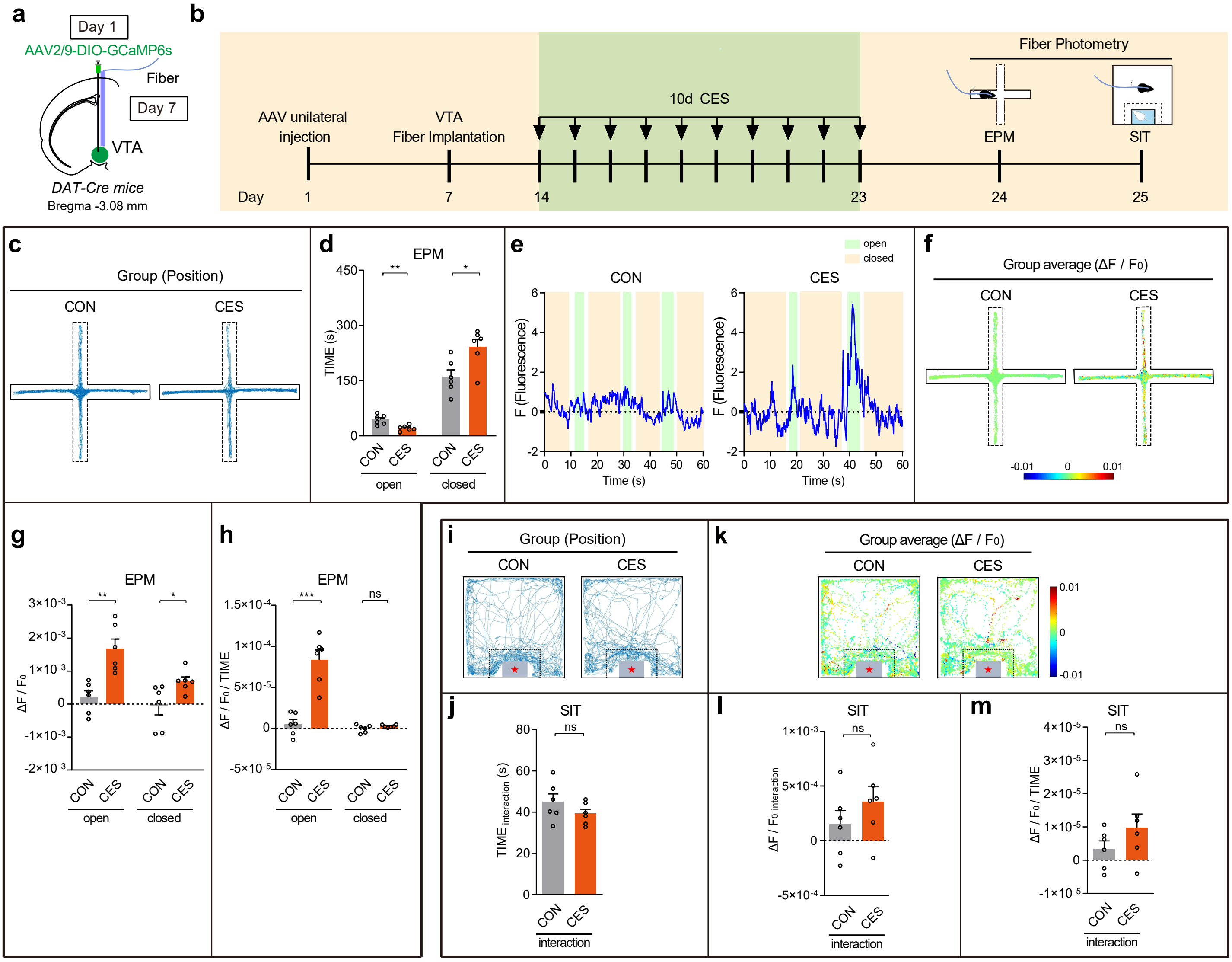

Supplementary Figure 5. VTA^{DA} neurons of CES mice showed significant activation of GCaMP6s activity in anxiety-inducing contexts.

(a) Schematic of GCaMP6s infection and optical fiber implantation in DAT-Cre mice. **(b)** Experimental scheme of CES modeling and position-synchronized in vivo calcium imaging in EPM. **c–h** Results of EPM. **(c)** Group of exploration traces in EPM. **(d)** Statistics of time spent in the open-arm and closed-arm of EPM (open arms: two-tailed unpaired t test, *P* = 0.0043; closed arms: two-tailed unpaired t test, *P* = 0.0174). **(e)** The representative traces of Ca^{2+} fluorescence in CON and CES mouse. The transparent cyan background and papaya whip color display the mice located in the open arms and closed arms, respectively. **(f)** Group average of Ca^{2+} activities of VTA^{DA} neurons in EPM. **(g)** Statistics of average Ca^{2+} activities of VTA^{DA} neurons in the open-arm and closed-arm of EPM (open arms: two-tailed unpaired t test, *P* = 0.0019; closed arms: two-tailed unpaired t test, *P* = 0.0337). **(h)** Statistics of variation rate of Ca^{2+} fluorescence in the open-arm and closed-arm of EPM (open arms: two-tailed unpaired t test, $P = 1.39 \times 10^{-4}$; closed arms: two-tailed unpaired t test, *P* = 0.1458). **i–m** Results of SIT. **(i)** Group of exploration traces in SIT. **(j)** Statistics of time spent in the social interaction zone of SIT (two-tailed unpaired t test, *P* = 0.2196). **(k)** Group average of Ca^{2+} activities of VTA^{DA} neurons in SIT. **(l)** Statistics of average Ca^{2+} activities of VTA^{DA} neurons in the social interaction zone of SIT (two-tailed unpaired t test, *P* = 0.2988). **(m)** Statistics of variation rate of Ca^{2+} fluorescence in the social interaction zone of SIT (two-tailed unpaired t test, *P* = 0.2077). n = 6 mice for each group. All data are shown as mean ± SEM. ns, not significant, *$P \le 0.05$, **$P \le 0.01$, ***$P \le 0.001$.

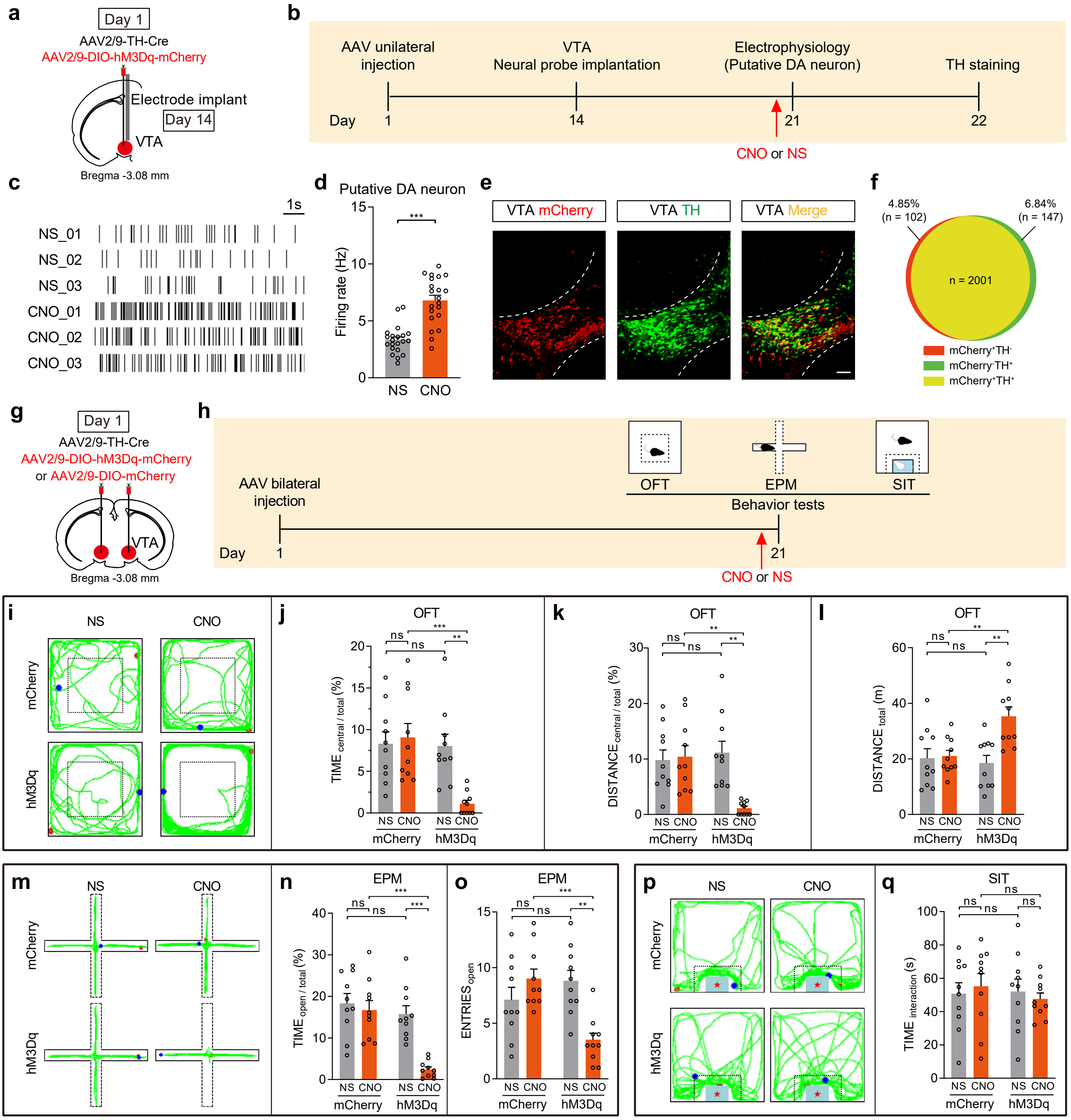

Supplementary Figure 6. Chemogenetic activation of VTA^{DA} neurons directly triggers anxiety-like behavior. a–f Verify the experimental validity of chemogenetic activation and *in vivo* electrophysiology recordings of VTA^{DA} neurons. **(a)** Schematic of virus infection and electrode implantation in VTA. **(b)** Experimental scheme of chemogenetic activation and *in vivo* electrophysiology recordings of VTA^{DA} neurons. **(c)** Raster plot of an hM3Dq-mCherry mouse after normal saline (NS) or CNO injection. **(d)** Firing rate of VTA^{DA} neurons following NS or CNO administration (two-tailed paired t test, $P = 4.92 \times 10^{-8}$). n = 21 neurons from 3 mice. **(e)** Representative image of VTA injection sites. Scale bar, 100 μm. **(f)** The quantitative analysis of Venn diagram shows the co-expression level of mCherry with TH in VTA (3 sections per mouse from 6 mice). **g–q** Bilateral activation of VTA^{DA} neurons by chemogenetic approach and behavioral tests (n = 10 mice for each group). **(g)** Schematic of bilateral virus infection. **(h)** Experimental scheme of chemogenetic activation of VTA^{DA} neurons and behavioral tests. **i–l** Results of OFT. **(i)** Representative exploration traces of OFT. **(j)** Percentage of time spent in the central area of OFT (two-way ANOVA, group: $P = 0.0042$, treatment: $P = 0.0273$, interaction: $P = 0.0070$; post hoc Tukey's test, hM3Dq (NS vs. CNO): $P = 0.0044$, CNO (mCherry vs. hM3Dq): $P = 0.0010$). **(k)** Percentage of distance traveled in the central area of OFT (two-way ANOVA, group: $P = 0.0275$, treatment: $P = 0.0106$, interaction: $P = 0.0042$; post hoc Tukey's test, hM3Dq (NS vs. CNO): $P = 0.0014$, CNO (mCherry vs. hM3Dq): $P = 0.0030$). **(l)** Total distance traveled in the OFT (two-way ANOVA, group: $P = 0.0438$, treatment: $P = 0.0056$, interaction: $P = 0.0109$; post hoc Tukey's test, hM3Dq (NS vs. CNO): $P = 0.0017$, CNO (mCherry vs. hM3Dq): $P = 0.0092$). **m–o** Results of EPM. **(m)** Representative exploration traces in EPM. **(n)** Percentage of time spent in the open arms of EPM (two-way ANOVA, group: $P = 0.0001$, treatment: $P = 0.0005$, interaction: $P = 0.0046$; post hoc Tukey's test, hM3Dq (NS vs. CNO): $P = 0.0001$, CNO (mCherry vs. hM3Dq): $P = 4.51 \times 10^{-5}$). **(o)** Number of open arm entries in the EPM (two-way ANOVA, group: $P = 0.0398$, treatment: $P = 0.0636$, interaction: $P = 0.0003$; post hoc Tukey's test, hM3Dq (NS vs. CNO): $P = 0.0010$, CNO (mCherry vs. hM3Dq): $P = 0.0006$). **p, q** Results of SIT. **(p)** Representative traces in SIT. **(q)** Statistics of time spent in the social interaction zone of SIT (two-way ANOVA, group: $P = 0.6478$, treatment: $P = 0.9953$, interaction: $P = 0.4929$; post hoc Tukey's test, hM3Dq(NS vs. CNO): $P = 0.9618$, CNO (mCherry vs. hM3Dq): $P = 0.8467$). The red and blue dots in the OFT, EPM and SIT locomotion traces reflect the start and end points of the mouse, respectively. All data are shown as mean ± SEM. ns, not significant, $^{**}P \le 0.01$, $^{***}P \le 0.001$.

a

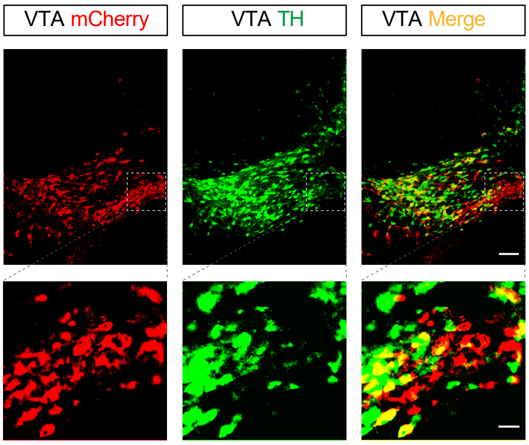

Supplementary Figure 7. Enlarged images of the labeled regions are shown to Supplementary Figure 6e.

(a) Enhanced images of the labeled regions in Supplementary Figure 6e are shown. The fluorescence signals are overexposed. Scale bar, 100 μm (top) and 25 μm (bottom).

a

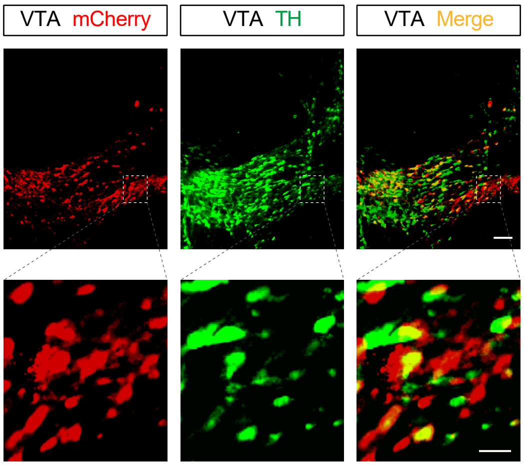

Supplementary Figure 8. Enlarged images of the labeled regions are shown to Fig. 4e.
(a) Enhanced images of the labeled regions in Fig. 4e are shown. The fluorescence signals are overexposed. Scale bar, 100 μm (top) and 25 μm (bottom).

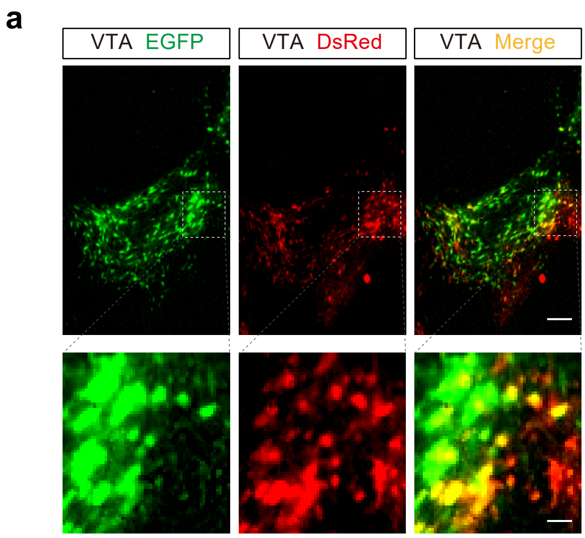

Supplementary Figure 9. Enlarged images of the labeled regions are shown to Fig. 6c.
(a) Enhanced images of the labeled regions in Fig. 6c are shown. The fluorescence signals are overexposed. Scale bar, 100 µm (top) and 25 µm (bottom).

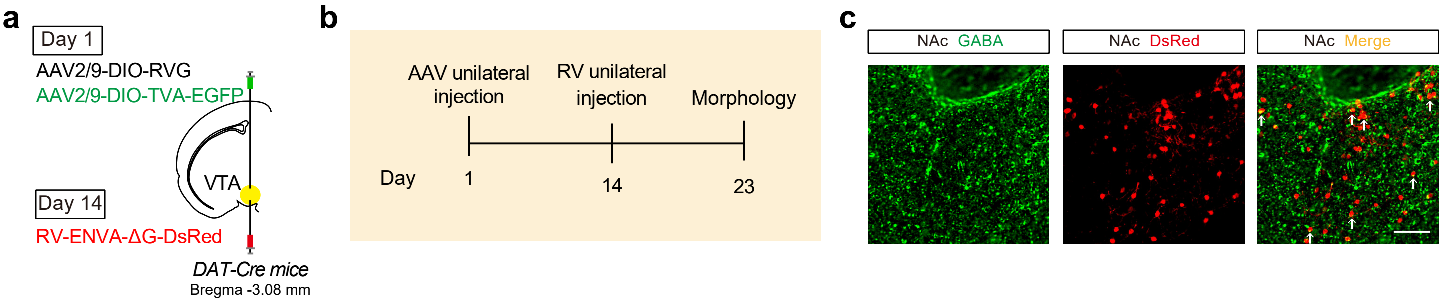

Supplementary Figure 10. NAc GABAergic neurons innervate VTA^{DA} neurons.

(a) Schematic of the Cre-dependent retrograde trans-monosynaptic rabies virus tracing strategy in DAT-Cre mice. **(b)** Experimental scheme of retrograde tracing. **(c)** DsRed-labeled neurons in the NAc traced from the VTA^{DA} neurons and DsRed signals were co-localized with GABAergic neuronal marker GABA immunofluorescence in the NAc (green, GABA; red, DsRed). The experiment was repeated three times with similar results. Scale bars, 100 μm.

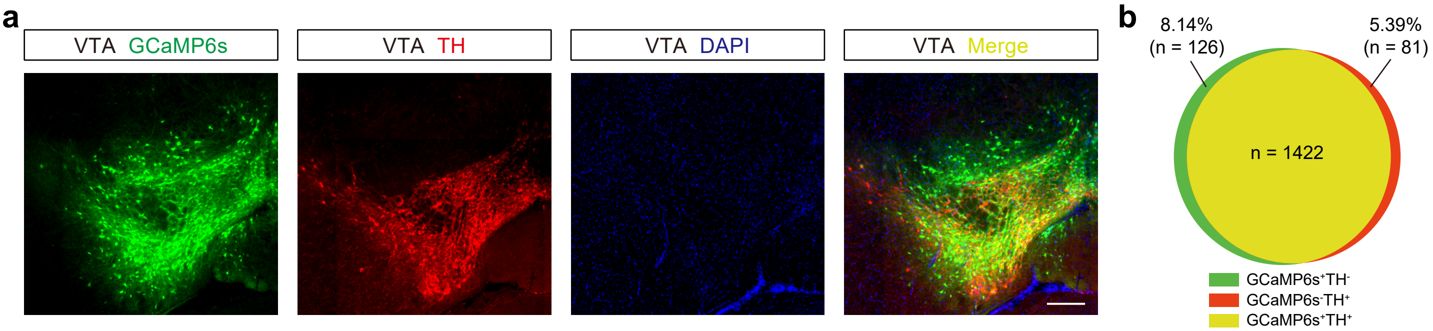

Supplementary Figure 11. Representative photographs and quantification show double staining of TH and GCaMP6s in the VTA (to Fig. 6o).

(a) Representative images show the co-expression of GCaMP6s with TH in VTA (green, GCaMP6s; red, TH; blue, DAPI). Scale bars, 100 μm. **(b)** The quantitative analysis of Venn diagram shows the co-expression level of GCaMP6s with TH in VTA (3 sections per mouse from 5 mice).

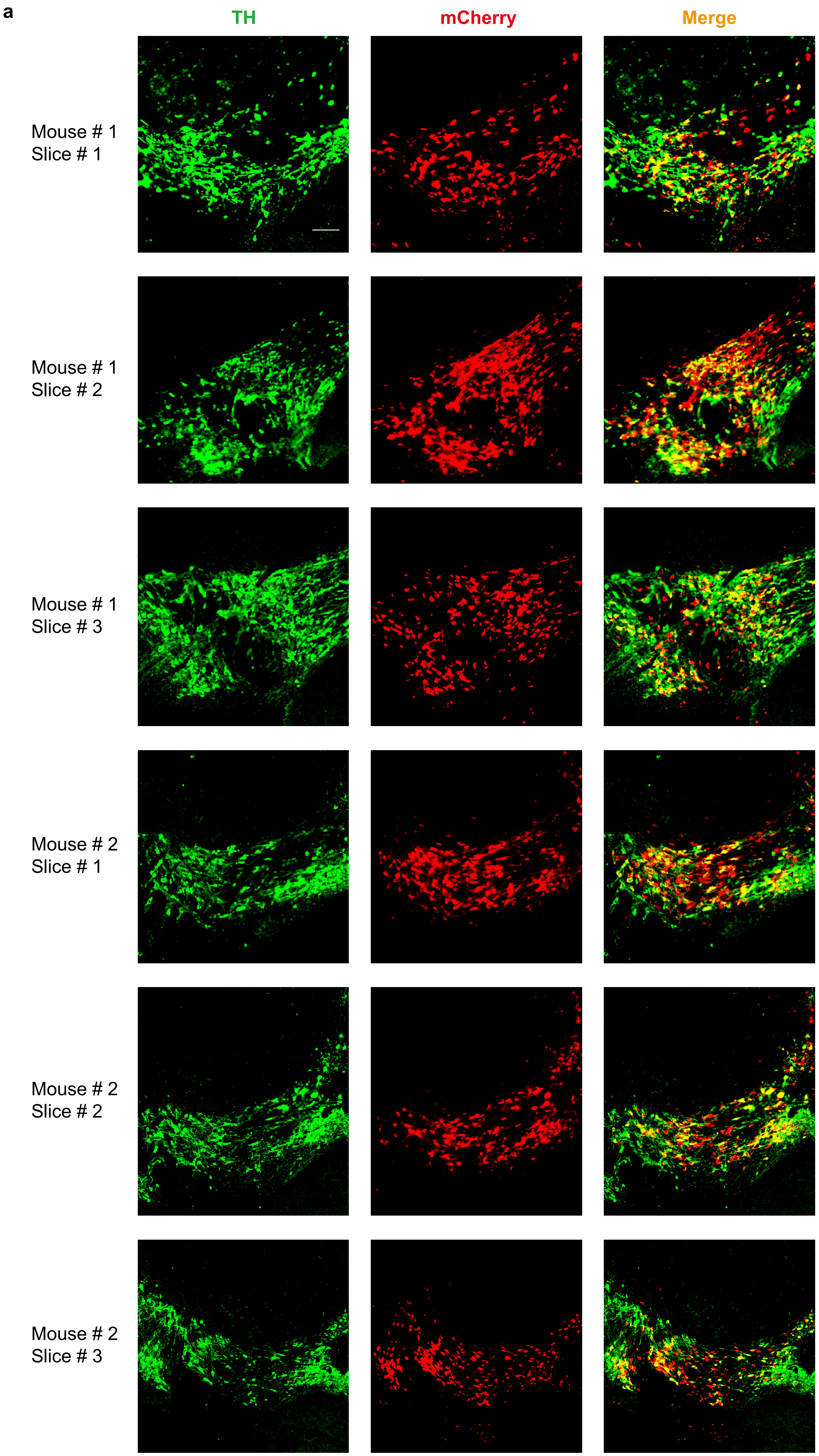

(Continued on next page)

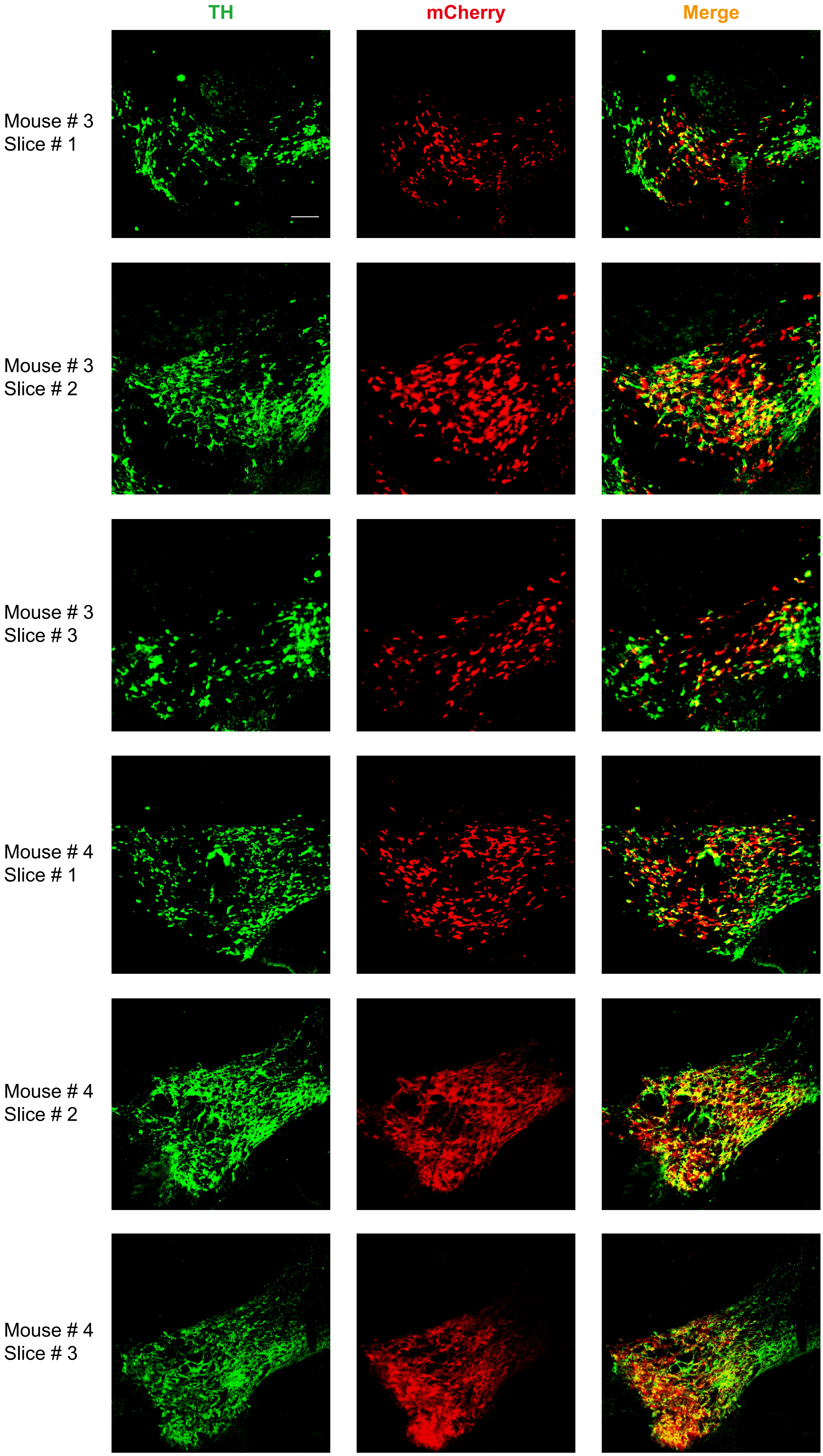

(Continued on next page)

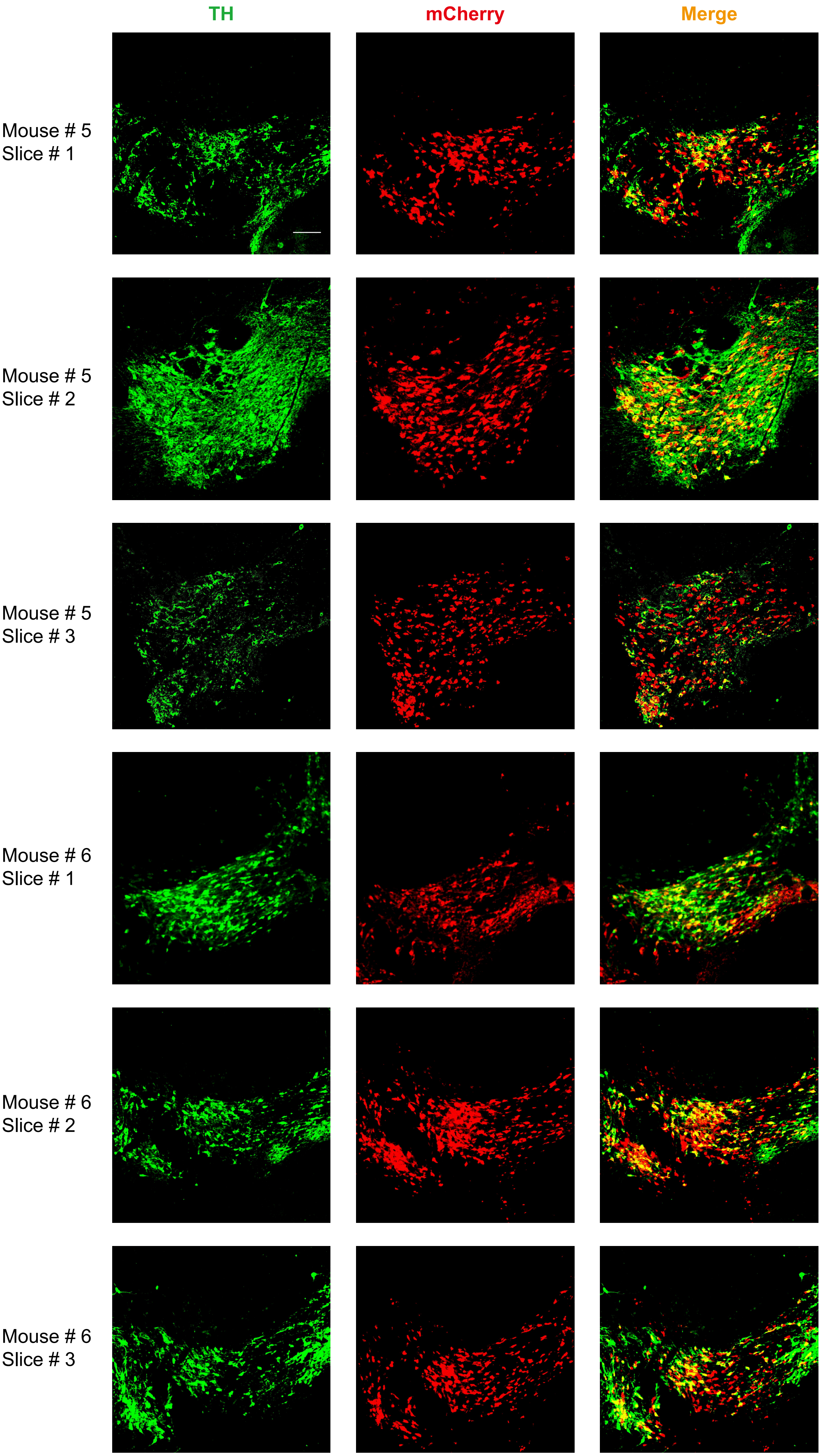

(Continued on next page)

b

Mouse #	Slice #	Cell counting		
		$mCherry^+$	TH^+	$mCherry^+TH^+$
1	1	115	119	114
	2	126	130	123
	3	134	140	132
2	1	109	115	102
	2	90	94	87
	3	86	89	78
3	1	119	113	110
	2	126	127	118
	3	112	108	99
4	1	116	118	115
	2	145	149	138
	3	135	136	125
5	1	118	121	116
	2	130	132	127
	3	115	121	105
6	1	120	122	114
	2	107	112	103
	3	100	102	95
	Total	***2103***	***2148***	***2001***

$$n_{mCherry+TH-} = n_{mCherry+} - n_{mCherry+TH+} = 2103 - 2001 = 102$$

$$P_{mCherry+TH-} = \frac{n_{mCherry+TH-}}{n_{mCherry+}} = \frac{102}{2103} = 4.85\%$$

$$n_{mCherry-TH+} = n_{TH+} - n_{mCherry+TH+} = 2148 - 2001 = 147$$

$$P_{mCherry-TH+} = \frac{n_{mCherry-TH+}}{n_{TH+}} = \frac{147}{2148} = 6.84\%$$

(Figure 3f)

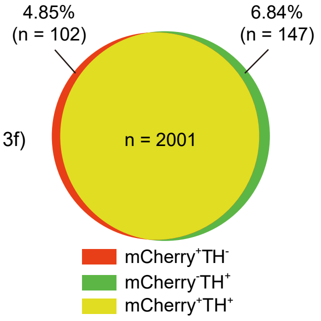

Supplementary Figure 12. The detailed histology data of Venn diagram (to Supplementary Figure. 6f).
(a) Fluorescence images showing double staining of mCherry with TH in the VTA region. The slice # 1 of mouse # 6 corresponds to the slice shown in Supplementary Figure 6e. Scale bar, 100 µm. **(b)** The quantitative analysis of the co-expression level of mCherry with TH in the VTA region. Venn diagram shows cells only positive for mCherry, cells only positive for TH, and double-positive cells. n, number; P, proportion.

Statistical Report

Figure number	n	Normality test	Equal variance test	Statistic method	*P* value	F/t value	Post hoc multiple comparisons test
1d<br>SIT: social interaction time	n=15 for CON<br>n=14 for CES<br>n=16 for CSDS	passed	passed	two-way RM ANOVA with Tukey's multiple comparisons test			group: F(2,42)=36.86, $P=5.71\times10^{-10}$<br>time: F(4,168)=3.827, *P*=0.0053<br>interaction: F(8,168)=2.098, *P*=0.0385<br>multiple comparisons:<br>day1: CON vs. CES: *P*=0.6937<br>CON vs. CSDS: *P*=0.6472<br>day3: CON vs. CES: *P*=0.0221<br>CON vs. CSDS: *P*=0.0004<br>day5: CON vs. CES: *P*=0.0018<br>CON vs. CSDS: *P*=0.0001<br>day7: CON vs. CES: *P*=0.0192<br>CON vs. CSDS: $P=6.10\times10^{-7}$<br>day9: CON vs. CES: *P*=0.2583,<br>CON vs. CSDS: $P=2.70\times10^{-6}$
1f<br>SIT: social interaction time<br>(Day 11)	n=15 for CON<br>n=14 for CES<br>n=16 for CSDS	failed		Kruskal-Wallis test	*P*=0.0005	H=15.2	CON vs. CES: *P*=0.9752<br>CON vs. CSDS: *P*=0.0005<br>CES vs. CSDS: *P*=0.0212
1h<br>OFT: central time	n=15 for CON<br>n=14 for CES<br>n=16 for CSDS	passed	passed	one-way ANOVA with Tukey's multiple comparisons test	$P=1.95\times10^{-6}$	$F_{2,42}=18.27$	CON vs. CES: $P=9.61\times10^{-6}$<br>CON vs. CSDS: $P=2.30\times10^{-5}$<br>CES vs. CSDS: *P*=0.8983
1i<br>OFT: central distance	n=15 for CON<br>n=14 for CES<br>n=16 for CSDS	passed	passed	one-way ANOVA with Tukey's multiple comparisons test	$P=3.16\times10^{-5}$	$F_{2,42}=13.4$	CON vs. CES: *P*=0.0001<br>CON vs. CSDS: *P*=0.0003<br>CES vs. CSDS: *P*=0.8992
1j<br>OFT: total distance	n=15 for CON<br>n=14 for CES<br>n=16 for CSDS	passed	passed	one-way ANOVA with Tukey's multiple comparisons test	$P=2.88\times10^{-5}$	$F_{2,42}=13.55$	CON vs. CES: *P*=0.0998<br>CON vs. CSDS: $P=1.76\times10^{-5}$<br>CES vs. CSDS: *P*=0.0144
1l<br>EPM: open arms time	n=15 for CON<br>n=14 for CES<br>n=16 for CSDS	passed	passed	one-way ANOVA with Tukey's multiple comparisons test	*P*=0.0016	$F_{2,42}=7.537$	CON vs. CES: *P*=0.0070<br>CON vs. CSDS: *P*=0.0032<br>CES vs. CSDS: *P*=0.9844
1m<br>EPM: number of entries into open arms	n=15 for CON<br>n=14 for CES<br>n=16 for CSDS	passed	passed	one-way ANOVA with Tukey's multiple comparisons test	*P*=0.1305	$F_{2,42}=2.139$	CON vs. CES: *P*=0.2197<br>CON vs. CSDS: *P*=0.1587<br>CES vs. CSDS: *P*=0.9919
1n<br>TST	n=15 for CON<br>n=14 for CES<br>n=16 for CSDS	passed	passed	one-way ANOVA with Tukey's multiple comparisons test	$P=9.91\times10^{-5}$	$F_{2,42}=11.58$	CON vs. CES: *P*=0.7461<br>CON vs. CSDS: *P*=0.0002<br>CES vs. CSDS: *P*=0.0020

1o<br>SPT: sucrose preference	n=15 for CON<br>n=14 for CES<br>n=16 for CSDS	passed	passed	one-way ANOVA with Tukey's multiple comparisons test	*P*=0.0014	$F_{2,42}$=7.675	CON vs. CES: *P*=0.8943<br>CON vs. CSDS: *P*=0.0024<br>CES vs. CSDS: *P*=0.0101
1p<br>SPT: total intake	n=15 for CON<br>n=14 for CES<br>n=16 for CSDS	passed	passed	one-way ANOVA with Tukey's multiple comparisons test	*P*=0.1401	$F_{2,42}$=2.06	CON vs. CES: *P*=0.9671<br>CON vs.CSDS: *P*=0.1603<br>CES vs.CSDS: *P*=0.2631
2c<br>c-fos	n=4 mice/group	failed		two-tailed unpaired t test with Welch's correction	*P*=0.0045		
2f<br>Firing rate	n=11 neurons<br>from 3 mice/group	passed	passed	two-tailed unpaired t test	*P*=0.5557		
2o<br>EPM: open arms time	n=7 mice/group	passed	passed	two-tailed unpaired t test	*P*=0.012		
2o<br>EPM: closed arms time	n=7 mice/group	passed	passed	two-tailed unpaired t test	*P*=0.0020		
2r<br>EPM: ΔF/F0 in open arms	n=7 mice/group	passed	passed	two-tailed unpaired t test	*P*=0.0062		
2r<br>EPM: ΔF/F0 in closed arms	n=7 mice/group	passed	passed	two-tailed unpaired t test	*P*=0.4331		
2s<br>EPM: ΔF/F1/TIME<br>in open arms	n=7 mice/group	passed	passed	two-tailed unpaired t test	*P*=0.0002		
2s<br>EPM: ΔF/F2/TIME<br>in closed arms	n=7 mice/group	passed	passed	two-tailed unpaired t test	*P*=0.5442		
2u<br>SIT: social interaction time	n=7 mice/group	passed	passed	two-tailed unpaired t test	*P*=0.1043		
2w<br>SIT: ΔF/F0	n=7 mice/group	passed	passed	two-tailed unpaired t test	*P*=0.8763		
2x<br>SIT: ΔF/F1/TIME	n=7 mice/group	passed	passed	two-tailed unpaired t test	*P*=0.8843		
3f<br>VTA-Gq: OFT-central time	n=8 mice/group	passed	passed	two-way ANOVA with Tukey's multiple comparisons test			group: F(1,28)=7.639, *P*=0.0100<br>treatment: F(1,28)=7.13, *P*=0.0125<br>interaction: F(1,28)=11.78, *P*=0.0019<br>multiple comparisons:<br>mCherry(NS vs. CNO): *P*=0.9488<br>hM3Dq(NS vs. CNO): *P*=0.0010<br>NS(mCherry vs. hM3Dq): *P*=0.9646<br>CNO(mCherry vs. hM3Dq): *P*=0.0008

3g<br>VTA-Gq: OFT-central distance	n=8 mice/group	passed	passed	two-way ANOVA with Tukey's multiple comparisons test	group: F(1,28)=9.97, *P*=0.0038<br>treatment: F(1,28)=8.532, *P*=0.0068<br>interaction: F(1,28)=5.207, *P*=0.0303<br>multiple comparisons:<br>mCherry(NS vs. CNO): *P*=0.9687<br>hM3Dq(NS vs. CNO): *P*=0.0052<br>NS(mCherry vs. hM3Dq): *P*=0.9251<br>CNO(mCherry vs. hM3Dq): *P*=0.0034
3h<br>VTA-Gq: OFT-total distance	n=8 mice/group	passed	passed	two-way ANOVA with Tukey's multiple comparisons test	group: F(1,28)=22.91, $P=4.97\times10^{-5}$<br>treatment: F(1,28)=29.23, $P=9.13\times10^{-6}$<br>interaction: F(1,28)=22.5, $P=5.60\times10^{-5}$<br>multiple comparisons:<br>mCherry(NS vs. CNO): *P*=0.9652<br>hM3Dq(NS vs. CNO): $P=4.78\times10^{-7}$<br>NS(mCherry vs. hM3Dq): *P*=0.9999<br>CNO(mCherry vs. hM3Dq): $P=1.49\times10^{-6}$
3j<br>VTA-Gq: EPM-open arms time	n=8 mice/group	passed	passed	two-way ANOVA with Tukey's multiple comparisons test	group: F(1,28)=12.6, *P*=0.0014<br>treatment: F(1,28)=13.67, *P*=0.0009<br>interaction: F(1,28)=10.38, *P*=0.0032<br>multiple comparisons:<br>mCherry(NS vs. CNO): *P*=0.9867<br>hM3Dq(NS vs. CNO): *P*=0.0002<br>NS(mCherry vs. hM3Dq): *P*=0.9955<br>CNO(mCherry vs. hM3Dq): *P*=0.0003
3k<br>VTA-Gq: EPM-number of entries into open arms	n=8 mice/group	passed	passed	two-way ANOVA with Tukey's multiple comparisons test	group: F(1,28)=3.972, *P*=0.0561<br>treatment: F(1,28)=0.4414, *P*=0.5119<br>interaction: F(1,28)=1.765, *P*=0.1947<br>multiple comparisons:<br>mCherry(NS vs. CNO): *P*=0.9651<br>hM3Dq(NS vs. CNO): *P*=0.5042<br>NS(mCherry vs. hM3Dq): *P*=0.9651<br>CNO(mCherry vs. hM3Dq): *P*=0.1111

3m<br>VTA-Gq: SI-social interaction time	n=8 mice/group	passed	passed	two-way ANOVA with Tukey's multiple comparisons test		group: F(1,28)=0.02959, *P*=0.8647<br>treatment: F(1,28)=0.2351, *P*=0.6315<br>interaction: F(1,28)=0.07535, *P*=0.7857<br>multiple comparisons:<br>mCherry(NS vs. CNO): *P*=0.9492<br>hM3Dq(NS vs. CNO): *P*=0.9988<br>NS(mCherry vs. hM3Dq): *P*=0.9889<br>CNO(mCherry vs. hM3Dq): *P*=0.9999
4d<br>Firing rate	n=16 neurons<br>from 3 mice	passed		two-tailed paired t test	*P*=0.0038	
4j<br>VTA-Gi: OFT-central time	n=10 mice/group	passed	passed	two-way ANOVA with Tukey's multiple comparisons test		group: F(1,36)=17.49, *P*=0.0002<br>treatment: F(1,36)=18.13, *P*=0.0001<br>interaction: F(1,36)=19.87, $P=7.80\times10^{-5}$<br>multiple comparisons:<br>mCherry(NS vs. CNO): *P*=0.9990<br>hM4Di(NS vs. CNO): $P=2.46\times10^{-6}$<br>NS(mCherry vs. hM4Di): *P*=0.9973<br>CNO(mCherry vs. hM4Di): $P=2.90\times10^{-6}$
4k<br>VTA-Gi: OFT-central distance	n=10 mice/group	passed	passed	two-way ANOVA with Tukey's multiple comparisons test		group: F(1,36)=5.469, *P*=0.0250<br>treatment: F(1,36)=9.915, *P*=0.0033<br>interaction: F(1,36)=8.016, *P*=0.0075<br>multiple comparisons:<br>mCherry(NS vs. CNO): *P*=0.9959<br>hM4Di(NS vs. CNO): *P*=0.0009<br>NS(mCherry vs. hM4Di): *P*=0.9852<br>CNO(mCherry vs. hM4Di): *P*=0.0043
4l<br>VTA-Gi: OFT-total distance	n=10 mice/group	passed	passed	two-way ANOVA with Tukey's multiple comparisons test		group: F(1,36)=0.8195, *P*=0.3714<br>treatment: F(1,36)=3.568E-06, *P*=0.9985<br>interaction: F(1,36)=0.8044, *P*=0.3757<br>multiple comparisons:<br>mCherry(NS vs. CNO): *P*=0.9198<br>hM4Di(NS vs. CNO): *P*=0.9207<br>NS(mCherry vs. hM4Di): *P*=0.9999<br>CNO(mCherry vs. hM4Di): *P*=0.5848

4n<br>VTA-Gi: EPM-open arms time	n=10 mice/group	passed	passed	two-way ANOVA with Tukey's multiple comparisons test	group: F(1,36)=6.879, *P*=0.0127<br>treatment: F(1,36)=8.858, *P*=0.0052<br>interaction: F(1,36)=8.181, *P*=0.0070<br>multiple comparisons:<br>mCherry(NS vs. CNO): *P*=0.9998<br>hM4Di(NS vs. CNO): *P*=0.0011<br>NS(mCherry vs. hM4Di): *P*=0.9983<br>CNO(mCherry vs. hM4Di): *P*=0.0023
4o<br>VTA-Gi: EPM-number of entries into open arms	n=10 mice/group	passed	passed	two-way ANOVA with Tukey's multiple comparisons test	group: F(1,36)=1.598, *P*=0.2143<br>treatment: F(1,36)=4.768, *P*=0.0356<br>interaction: F(1,36)=9.158, *P*=0.0046<br>multiple comparisons:<br>mCherry(NS vs. CNO): *P*=0.9326<br>hM4Di(NS vs. CNO): *P*=0.0040<br>NS(mCherry vs. hM4Di): *P*=0.6023<br>CNO(mCherry vs. hM4Di): *P*=0.0221
4q<br>VTA-Gi: SI-social interaction time	n=10 mice/group	passed	passed	two-way ANOVA with Tukey's multiple comparisons test	group: F(1,36)=0.1661, *P*=0.6860<br>treatment: F(1,36)=0.05331, *P*=0.8187<br>interaction: F(1,36)=0.1319, *P*=0.7186<br>multiple comparisons:<br>mCherry(NS vs. CNO): *P*=0.9747<br>hM4Di(NS vs. CNO): *P*=0.9997<br>NS(mCherry vs. hM4Di): *P*=0.9473<br>CNO(mCherry vs. hM4Di): *P*=0.9999
5d<br>OFT: central time	n=9 for mCherry-NS<br>n=9 for mCherry-CNO<br>n=8 for hM4Di-NS<br>n=8 for hM4Di-CNO	passed	passed	two-way ANOVA with Tukey's multiple comparisons test	group: F(1,30)=8.739, *P*=0.0060<br>treatment: F(1,30)=10.17, P=0.0033<br>interaction: F(1,30)=6.788, P=0.0141<br>multiple comparisons:<br>mCherry(NS vs. CNO): P=0.9738<br>hM4Di(NS vs. CNO): P=0.0022<br>NS(mCherry vs. hM4Di): P=0.9945<br>CNO(mCherry vs. hM4Di): P=0.0025

5e<br>OFT: central distance	n=9 for mCherry-NS<br>n=9 for mCherry-CNO<br>n=8 for hM4Di-NS<br>n=8 for hM4Di-CNO	passed	passed	two-way ANOVA with Tukey's multiple comparisons test	group: F(1,30)=4.872, *P*=0.0351<br>treatment: F(1,30)=5.023, *P*=0.0326<br>interaction: F(1,30)=3.316, *P*=0.0786<br>multiple comparisons:<br>mCherry(NS vs. CNO): *P*=0.9898<br>hM4Di(NS vs. CNO): *P*=0.0425<br>NS(mCherry vs. hM4Di): *P*=0.9927<br>CNO(mCherry vs. hM4Di): *P*=0.0373
5f<br>OFT: total distance	n=9 for mCherry-NS<br>n=9 for mCherry-CNO<br>n=8 for hM4Di-NS<br>n=8 for hM4Di-CNO	passed	passed	two-way ANOVA with Tukey's multiple comparisons test	group: F(1,30)=2.212, *P*=0.1474<br>treatment: F(1,30)=0.07345, *P*=0.3982<br>interaction: F(1,30)=2.283, *P*=0.1413<br>multiple comparisons:<br>mCherry(NS vs. CNO): *P*=0.9637<br>hM4Di(NS vs. CNO): *P*=0.3792<br>NS(mCherry vs. hM4Di): *P*=0.9999<br>CNO(mCherry vs. hM4Di): *P*=0.1699
5h<br>EPM: open arms time	n=9 for mCherry-NS<br>n=9 for mCherry-CNO<br>n=8 for hM4Di-NS<br>n=8 for hM4Di-CNO	passed	passed	two-way ANOVA with Tukey's multiple comparisons test	group: F(1,30)=7.05, *P*=0.0126<br>treatment: F(1,30)=10.16, *P*=0.0033<br>interaction: F(1,30)=9.051, *P*=0.0053<br>multiple comparisons:<br>mCherry(NS vs. CNO): *P*=0.9992<br>hM4Di(NS vs. CNO): *P*=0.0010<br>NS(mCherry vs. hM4Di): *P*=0.9944<br>CNO(mCherry vs. hM4Di): *P*=0.0020
5i<br>EPM: number of entries into open arms	n=9 for mCherry-NS<br>n=9 for mCherry-CNO<br>n=8 for hM4Di-NS<br>n=8 for hM4Di-CNO	passed	passed	two-way ANOVA with Tukey's multiple comparisons test	group: F(1,30)=0.5217, *P*=0.4757<br>treatment: F(1,30)=0.5577, *P*=0.4610<br>interaction: F(1,30)=0.2771, *P*=0.6025<br>multiple comparisons:<br>mCherry(NS vs. CNO): *P*=0.7901<br>hM4Di(NS vs. CNO): *P*=0.9987<br>NS(mCherry vs. hM4Di): *P*=0.9990<br>CNO(mCherry vs. hM4Di): *P*=0.8136

5k<br>SIT: interactional time	n=9 for mCherry-NS<br>n=9 for mCherry-CNO<br>n=8 for hM4Di-NS<br>n=8 for hM4Di-CNO	passed	passed	two-way ANOVA with Tukey's multiple comparisons test		group: $F(1,30)=0.01314$, $P=0.9095$<br>treatment: $F(1,30)=0.09247$, $P=0.7632$<br>interaction: $F(1,30)=0.7984$, $P=0.3787$<br>multiple comparisons:<br>mCherry(NS vs. CNO): $P=0.9729$<br>hM4Di(NS vs. CNO): $P=0.8431$<br>NS(mCherry vs. hM4Di): $P=0.8912$<br>CNO(mCherry vs. hM4Di): $P=0.9456$
6e<br>quantification of VTA DA neuron inputs	n=3 mice/group	passed	passed	two-way ANOVA with uncorrected Fisher's LSD		group: $F(1,48)=0.07062$, $P=0.7916$<br>region: $F(11,48)=37.73$, $P=1.00\times10^{-15}$<br>interaction: $F(11,48)=1.352$, $P=0.2266$<br>multiple comparisons:<br>MO: $P=0.4351$. VO: $P=0.9005$.<br>LO: $P=0.5135$. DLO: $P=0.7896$.<br>FrA: $P=$ 0.1153. PrL: $P=0.8681$.<br>M1: $P=0.2274$. M2: $P=0.4620$.<br>AI: $P=0.8618$. Cpu: $P=0.0569$.<br>NAc: $P=0.0256$. Cg1: $P=0.9153$.
6q<br>EPM: open arms time	n=7 mice/group	passed	passed	two-tailed unpaired t test	$P=0.0008$	
6q<br>EPM: closed arms time	n=7 mice/group	passed	passed	two-tailed unpaired t test	$P=0.0014$	
6t<br>EPM: ΔF/F0 in open arms	n=7 mice/group	passed	passed	two-tailed unpaired t test	$P=0.0025$	
6t<br>EPM: ΔF/F0 in closed arms	n=7 mice/group	passed	passed	two-tailed unpaired t test	$P=0.3500$	
6u<br>EPM: ΔF/F0/TIME<br>in open arms	n=7 mice/group	passed	passed	two-tailed unpaired t test	$P=0.0006$	
6u<br>EPM: ΔF/F0/TIME<br>in closed arms	n=7 mice/group	passed	passed	two-tailed unpaired t test	$P=0.3182$	

7f<br>NAC-VTA: OFT-central time	n=8 mice/group	passed	passed	two-way ANOVA with Bonferroni's multiple comparisons test	group: F(1,28)=9.339, *P*=0.0049<br>laser: F(1,28)=6.163, *P*=0.0193<br>interaction: F(1,28)=8.908, *P*=0.0058<br>multiple comparisons:<br>mCherry(off vs. on): *P*=0.9999<br>NpHR(off vs. on): *P*=0.0036<br>Off(mCherry vs. NpHR): *P*=0.9999<br>On(mCherry vs. NpHR): *P*=0.0012
7g<br>NAC-VTA: OFT-central distance	n=8 mice/group	passed	passed	two-way ANOVA with Bonferroni's multiple comparisons test	group: F(1,28)=12.25, *P*=0.0016<br>laser: F(1,28)=5.081, *P*=0.0322<br>interaction: F(1,28)=8.177, *P*=0.0079<br>multiple comparisons:<br>mCherry(off vs. on): *P*=0.9999<br>NpHR(off vs. on): *P*=0.0070<br>Off(mCherry vs. NpHR): *P*=0.9999<br>On(mCherry vs. NpHR): *P*=0.0007
7h<br>NAC-VTA: OFT-total distance	n=8 mice/group	passed	passed	two-way ANOVA with Bonferroni's multiple comparisons test	group: F(1,28)=3.01, *P*=0.0938<br>laser: F(1,28)=0.36, *P*=0.5533<br>interaction: F(1,28)=0.1166, *P*=0.7353<br>multiple comparisons:<br>mCherry(off vs. on): *P*=0.9999<br>NpHR(off vs. on): *P*=0.9999<br>Off(mCherry vs. NpHR): *P*=0.9999<br>On(mCherry vs. NpHR): *P*=0.9193
7j<br>NAC-VTA: EPT-open arms time	n=8 mice/group	passed	passed	two-way ANOVA with Bonferroni's multiple comparisons test	group: F(1,28)=6.356, *P*=0.0177<br>laser: F(1,28)=12.07, *P*=0.0017<br>interaction: F(1,28)=7.273, *P*=0.0117<br>multiple comparisons:<br>mCherry(off vs. on): *P*=0.9999<br>NpHR(off vs. on): *P*=0.0009<br>Off(mCherry vs. NpHR): *P*=0.9999<br>On(mCherry vs. NpHR): *P*=0.0058

7k<br>NAC-VTA: EPT-number of entries into open arms	n=8 mice/group	passed	passed	two-way ANOVA with Bonferroni's multiple comparisons test		group: F(1,28)=1.079, *P*=0.3078<br>laser: F(1,28)=1.918, *P*=0.1770<br>interaction: F(1,28)=1.918, *P*=0.1770<br>multiple comparisons:<br>mCherry(off vs. on): *P*=0.9999<br>NpHR(off vs. on): *P*=0.3611<br>Off(mCherry vs. NpHR): *P*=0.9999<br>On(mCherry vs. NpHR): *P*=0.5857
7o<br>NAC-VTA: OFT-central time	n=10 mice/group	passed	passed	two-tailed unpaired t test	*P*=0.0018	
7p<br>NAC-VTA: OFT-central distance	n=10 mice/group	passed	passed	two-tailed unpaired t test	*P*=0.0052	
7q<br>NAC-VTA: OFT-total distance	n=10 mice/group	passed	passed	two-tailed unpaired t test	*P*=0.2154	
7s<br>NAC-VTA: EPT-open arms time	n=10 mice/group	passed	passed	two-tailed unpaired t test	*P*=0.0005	
7t<br>NAC-VTA: EPT-number of entries into open arms	n=10 mice/group	passed	passed	two-tailed unpaired t test	*P*=0.0018	
8d<br>AAV1: OFT-central time	n=8 mice/group	passed	passed	two-way ANOVA with Tukey's multiple comparisons test		group: F(1,28)=5.818, *P*=0.0227<br>laser: F(1,28)=4.29, *P*=0.0477<br>interaction: F(1,28)=7.534, *P*=0.0104<br>multiple comparisons:<br>mCherry(off vs. on): *P*=0.9637<br>NpHR(off vs. on): *P*=0.0102<br>Off(mCherry vs. NpHR): *P*=0.9953<br>On(mCherry vs. NpHR): *P*=0.0056
8e<br>AAV1: OFT-central distance	n=8 mice/group	passed	passed	two-way ANOVA with Tukey's multiple comparisons test		group: F(1,28)=5.568, *P*=0.0255<br>laser: F(1,28)=4.483, *P*=0.0432<br>interaction: F(1,28)=4.175, *P*=0.0505<br>multiple comparisons:<br>mCherry(off vs. on): *P*=0.9999<br>NpHR(off vs. on): *P*=0.0310<br>Off(mCherry vs. NpHR): *P*=0.9960<br>On(mCherry vs. NpHR): *P*=0.0208

8f<br>AAV1: OFT-total distance	n=8 mice/group	passed	passed	two-way ANOVA with Tukey's multiple comparisons test		group: F(1,28)=0.6031, *P*=0.4439<br>laser: F(1,28)=0.03545, *P*=0.8520<br>interaction: F(1,28)=0.5703, *P*=0.4565<br>multiple comparisons:<br>mCherry(off vs. on): *P*=0.9778<br>NpHR(off vs. on): *P*=0.9086<br>Off(mCherry vs. NpHR): *P*=0.7025<br>On(mCherry vs. NpHR): *P*=0.9999
8h<br>AAV1: EPM-open arms time	n=8 mice/group	passed	passed	two-way ANOVA with Tukey's multiple comparisons test		group: F(1,28)=4.328, *P*=0.0468<br>laser: F(1,28)=9.642, *P*=0.0043<br>interaction: F(1,28)=4.244, *P*=0.0488<br>multiple comparisons:<br>mCherry(off vs. on): *P*=0.8805<br>NpHR(off vs. on): *P*=0.0055<br>Off(mCherry vs. NpHR): *P*=0.9999<br>On(mCherry vs. NpHR): *P*=0.0321
8i<br>AAV1: EPM-number of entries into open arms	n=8 mice/group	passed	passed	two-way ANOVA with Tukey's multiple comparisons test		group: F(1,28)=3.073, *P*=0.0906<br>laser: F(1,28)=1.897, *P*=0.1793<br>interaction: F(1,28)=1.897, *P*=0.1793<br>multiple comparisons:<br>mCherry(off vs. on): *P*=0.9999<br>NpHR(off vs. on): *P*=0.2318<br>Off(mCherry vs. NpHR): *P*=0.9933<br>On(mCherry vs. NpHR): *P*=0.1443
8m<br>AAV1: OFT-central time	n=8 mice/group	passed	passed	two-tailed unpaired t test	*P*=0.0009	
8n<br>AAV1: OFT-central distance	n=8 mice/group	passed	passed	two-tailed unpaired t test	*P*=0.0008	
8o<br>AAV1: OFT-total distance	n=8 mice/group	passed	passed	two-tailed unpaired t test	*P*=0.9683	
8q<br>AAV1: EPM-open arms time	n=8 mice/group	passed	passed	two-tailed unpaired t test	*P*=0.0075	

8r<br>AAV1: EPM-number<br>of entries into open arms	n=8 mice/group	passed	passed	two-tailed unpaired t test	*P*=0.8755		
S1a<br>SIT: total distance	n=15 for CON<br>n=14 for CES<br>n=16 for CSDS	passed	passed	two-way RM ANOVA with Tukey's<br>multiple comparisons test			group: F(2,42)=50.72, $P=6.29\times10^{-12}$<br>time: F(4,168)=4.628, *P*=0.0014<br>interaction: F(8,168)=3.445, *P*=0.0011<br>multiple comparisons:<br>day1: CON vs. CES: *P*=0.9999<br>CON vs. CSDS: *P*=0.3649<br>day3: CON vs. CES: *P*=0.0025<br>CON vs. CSDS: *P*=0.0009<br>day5: CON vs. CES: $P=7.05\times10^{-6}$<br>CON vs. CSDS: $P=2.77\times10^{-6}$<br>day7: CON vs. CES: $P=2.96\times10^{-7}$<br>CON vs. CSDS: $P=6.85\times10^{-9}$<br>day9: CON vs. CES: *P*=0.0047<br>CON vs. CSDS: $P=7.72\times10^{-9}$
S1b<br>SIT: total distance<br>on day 11	n=15 for CON<br>n=14 for CES<br>n=16 for CSDS	passed	passed	one-way ANOVA with Tukey's<br>multiple comparisons test	$P=5.30\times10^{-6}$	$F_{2,42}=16.45$	CON vs. CES: *P*=0.1219<br>CON vs. CSDS: $P=3.64\times10^{-6}$<br>CES vs. CSDS: *P*=0.0030
S2a<br>number of c-fos<br>in RN	n=4 mice/group	failed		two-tailed Mann Whitney test	*P*=0.0286		
S5d<br>EPM: open arms time	n=6 mice/group	passed	passed	two-tailed unpaired t test	*P*=0.0043		
S5d<br>EPM: closed arms time	n=6 mice/group	passed	passed	two-tailed unpaired t test	*P*=0.0174		
S5g<br>EPM: ΔF/F0 in open arms	n=6 mice/group	passed	passed	two-tailed unpaired t test	*P*=0.0019		
S5g<br>EPM: ΔF/F0 in closed arms	n=6 mice/group	passed	passed	two-tailed unpaired t test	*P*=0.0337		
S5h<br>EPM: ΔF/F1/TIME<br>in open arms	n=6 mice/group	passed	passed	two-tailed unpaired t test	$P=1.39\times10^{-4}$		
S5h<br>EPM: ΔF/F2/TIME<br>in closed arms	n=6 mice/group	passed	passed	two-tailed unpaired t test	*P*=0.1458		

S5j<br>SIT: social interaction time	n=6 mice/group	passed	passed	two-tailed unpaired t test	*P*=0.2196	
S5l<br>SIT: ΔF/F0	n=6 mice/group	passed	passed	two-tailed unpaired t test	*P*=0.2988	
S5m<br>SIT: ΔF/F1/TIME	n=6 mice/group	passed	passed	two-tailed unpaired t test	*P*=0.2077	
S6d<br>Firing rate	n=21 neurons<br>from 3 mice	passed		two-tailed paired t test	*P*=4.92×10^{-8}	
S6j<br>VTA-Gq: OFT-central time	n=10 mice/group	passed	passed	two-way ANOVA with Tukey's multiple comparisons test		group: F(1,36)=9.341, *P*=0.0042<br>treatment: F(1,36)=5.298, *P*=0.0273<br>interaction: F(1,36)=8.178, *P*=0.0070<br>multiple comparisons:<br>mCherry(NS vs. CNO): *P*=0.9788<br>hM3Dq(NS vs. CNO): *P*=0.0044<br>NS(mCherry vs. hM3Dq): *P*=0.9990<br>CNO(mCherry vs. hM3Dq): *P*=0.0010
S6k<br>VTA-Gq: OFT-central distance	n=10 mice/group	passed	passed	two-way ANOVA with Tukey's multiple comparisons test		group: F(1,36)=5.277, *P*=0.0275<br>treatment: F(1,36)=7.261, *P*=0.0106<br>interaction: F(1,36)=9.341, *P*=0.0042<br>multiple comparisons:<br>mCherry(NS vs. CNO): *P*=0.9940<br>hM3Dq(NS vs. CNO): *P*=0.0014<br>NS(mCherry vs. hM3Dq): *P*=0.9495<br>CNO(mCherry vs. hM3Dq): P=0.0030
S6l<br>VTA-Gq: OFT-total distance	n=10 mice/group	passed	passed	two-way ANOVA with Tukey's multiple comparisons test		group: F(1,36)=4.366, *P*=0.0438<br>treatment: F(1,36)=8.693, *P*=0.0056<br>interaction: F(1,36)=7.209, *P*=0.0109<br>multiple comparisons:<br>mCherry(NS vs. CNO): *P*=0.9977<br>hM3Dq(NS vs. CNO): *P*=0.0017<br>NS(mCherry vs. hM3Dq): *P*=0.9745<br>CNO(mCherry vs. hM3Dq): *P*=0.0092

S6n<br>VTA-Gq: EPM-open arms time	n=10 mice/group	passed	passed	two-way ANOVA with Tukey's multiple comparisons test	group: F(1,36)=18.91, *P*=0.0001<br>treatment: F(1,36)=14.77, *P*=0.0005<br>interaction: F(1,36)=9.124, *P*=0.0046<br>multiple comparisons:<br>mCherry(NS vs. CNO): *P*=0.9369<br>hM3Dq(NS vs. CNO): *P*=0.0001<br>NS(mCherry vs. hM3Dq): *P*=0.7840<br>CNO(mCherry vs. hM3Dq): $P=4.51\times10^{-5}$
S6o<br>VTA-Gq: EPM-number of entries into open arms	n=10 mice/group	passed	passed	two-way ANOVA with Tukey's multiple comparisons test	group: F(1,36)=4.549, *P*=0.0398<br>treatment: F(1,36)=3.664, *P*=0.0636<br>interaction: F(1,36)=15.94, *P*=0.0003<br>multiple comparisons:<br>mCherry(NS vs. CNO): *P*=0.4659<br>hM3Dq(NS vs. CNO): *P*=0.0010<br>NS(mCherry vs. hM3Dq): *P*=0.5597<br>CNO(mCherry vs. hM3Dq): *P*=0.0006
S6q<br>VTA-Gq: SI-social interaction time	n=10 mice/group	passed	passed	two-way ANOVA with Tukey's multiple comparisons test	group: F(1,36)=0.2123, *P*=0.6478<br>treatment: $F(1,36)=3.52\times10^{-5}$, *P*=0.9953<br>interaction: F(1,36)=0.48, *P*=0.4929<br>multiple comparisons:<br>mCherry(NS vs. CNO): *P*=0.9599<br>hM3Dq(NS vs. CNO): *P*=0.9618<br>NS(mCherry vs. hM3Dq): *P*=0.9984<br>CNO(mCherry vs. hM3Dq): *P*=0.8467
