# Supplementary material for: NAc-VTA circuit underlies emotional stress-induced anxiety-like behavior in the three-chamber vicarious social defeat stress mouse model
Source: Nat Commun. 2022 Jan 31;13:577. doi: 10.1038/s41467-022-28190-2 (PMC8804001; doi:10.1038/s41467-022-28190-2)
Supplement: Supplementary file 3 — Reporting Summary [file 41467_2022_28190_MOESM3_ESM.pdf]

nature portfolio

Corresponding author(s): Jie Ming
Bo Tian

Last updated by author(s): Dec 3, 2021

Reporting Summary

Nature Portfolio wishes to improve the reproducibility of the work that we publish. This form provides structure for consistency and transparency in reporting. For further information on Nature Portfolio policies, see our Editorial Policies and the Editorial Policy Checklist.

Statistics

For all statistical analyses, confirm that the following items are present in the figure legend, table legend, main text, or Methods section.

n/a	Confirmed	
☐	☒	The exact sample size (n) for each experimental group/condition, given as a discrete number and unit of measurement
☐	☒	A statement on whether measurements were taken from distinct samples or whether the same sample was measured repeatedly
☐	☒	The statistical test(s) used AND whether they are one- or two-sided<br>*Only common tests should be described solely by name; describe more complex techniques in the Methods section.*
☒	☐	A description of all covariates tested
☐	☒	A description of any assumptions or corrections, such as tests of normality and adjustment for multiple comparisons
☐	☒	A full description of the statistical parameters including central tendency (e.g. means) or other basic estimates (e.g. regression coefficient) AND variation (e.g. standard deviation) or associated estimates of uncertainty (e.g. confidence intervals)
☐	☒	For null hypothesis testing, the test statistic (e.g. F, t, r) with confidence intervals, effect sizes, degrees of freedom and P value noted<br>*Give P values as exact values whenever suitable.*
☒	☐	For Bayesian analysis, information on the choice of priors and Markov chain Monte Carlo settings
☒	☐	For hierarchical and complex designs, identification of the appropriate level for tests and full reporting of outcomes
☒	☐	Estimates of effect sizes (e.g. Cohen's d, Pearson's r), indicating how they were calculated

Our web collection on statistics for biologists contains articles on many of the points above.

Software and code

Policy information about availability of computer code

Data collection	For behavioral test(OFT, EPM, SIT and TST), SuperMaze+ XinRuan software was used. Electrophysiology data were acquired using Zeus-128. For fiber Photometry, data were acquired with Inper. For slice imaging, data were acquired with Olympus VS120.
Data analysis	Electrophysiology data were analyzed by NeuroExplorer 5. Fiber photometry data were analyzed by MATLAB R2018a. Fluorescence images were analyzed by Image J 1.53e. Data were graphed and analyzed using Graphpad Prism 7.

For manuscripts utilizing custom algorithms or software that are central to the research but not yet described in published literature, software must be made available to editors and reviewers. We strongly encourage code deposition in a community repository (e.g. GitHub). See the Nature Portfolio guidelines for submitting code & software for further information.

Data

Policy information about availability of data

All manuscripts must include a data availability statement. This statement should provide the following information, where applicable:

- Accession codes, unique identifiers, or web links for publicly available datasets
- A description of any restrictions on data availability
- For clinical datasets or third party data, please ensure that the statement adheres to our policy

The data supporting the findings of this study are available within the Article and Supplementary Information files or available from the corresponding author upon reasonable request. The source data generated in this study are provided in the Source Data file.

Field-specific reporting

Please select the one below that is the best fit for your research. If you are not sure, read the appropriate sections before making your selection.

☒ Life sciences ☐ Behavioural & social sciences ☐ Ecological, evolutionary & environmental sciences

For a reference copy of the document with all sections, see nature.com/documents/nr-reporting-summary-flat.pdf

Life sciences study design

All studies must disclose on these points even when the disclosure is negative.

Sample size	No statistical methods were used to pre-determine sample sizes. The number of animals in each group was determined according to previous study (Juarez B. et al., 2017). Samples sizes adopted in this study were sufficient for detecting robust effect. Sample sizes are indicated in the legends of Figures and supplementary Figures.
Data exclusions	No data was excluded from the study.
Replication	To ensure reproducibility, all experiments were performed at least three independent times using similar conditions. All attempts at replication were successful.
Randomization	The mice were randomized to put into separate groups /cages for allocating mice to different interventions.
Blinding	The investigators were blinded to group allocation during data collection and blinded to the treatment of mice to which mice were subjected when assessing animal behavior, fiber photometry and electrophysiology.

Reporting for specific materials, systems and methods

We require information from authors about some types of materials, experimental systems and methods used in many studies. Here, indicate whether each material, system or method listed is relevant to your study. If you are not sure if a list item applies to your research, read the appropriate section before selecting a response.

Materials & experimental systems

n/a	Involved in the study	
☐	☒	Antibodies
☒	☐	Eukaryotic cell lines
☒	☐	Palaeontology and archaeology
☐	☒	Animals and other organisms
☒	☐	Human research participants
☒	☐	Clinical data
☒	☐	Dual use research of concern

Methods

n/a	Involved in the study	
☒	☐	ChIP-seq
☒	☐	Flow cytometry
☒	☐	MRI-based neuroimaging

Antibodies

Antibodies used	Primary antibodies used:<br>Rabbit monoclonal anti-c-fos (9F6): CST, Cat. #2250S, Lot: 12. For IF - 1:1000<br>Rabbit polyclonal anti-TH: proteintech, Cat. 25859-1-AP, Lot: 00095148. For IF - 1:500<br>Rabbit polyclonal anti-GABA: Sigma, Cat. A2052, Lot: 038M4888V. For IF - 1:100<br>Secondary antibodies used:<br>Goat anti-rabbit, Alexa-488: Jackson ImmunoResearch, code: 111-545-144, 1:500<br>Goat anti-rabbit, Cy™3: Jackson ImmunoResearch, code: 111-165-144, 1:500
Validation	The specificity and application of all the antibodies are validated by companies.<br>Rabbit monoclonal anti-c-fos (9F6): CST, Cat.2250S, IF<br>Validation for IF- https://www.cellsignal.com/products/primary-antibodies/c-fos-9f6-rabbit-mab/2250<br>Rabbit polyclonal anti-TH: proteintech, Cat. 25859-1-AP, IF<br>Validation for IF - https://www.ptgcn.com/products/TH-Antibody-25859-1-AP.htm<br>Rabbit polyclonal anti-GABA: Sigma, Cat. A2052, IF<br>Validation for IF - https://www.sigmaaldrich.cn/CN/en/product/sigma/a2052?context=product

Animals and other organisms

Policy information about studies involving animals; ARRIVE guidelines recommended for reporting animal research

Laboratory animals	C57BL/6J mice (6-8 weeks, male), DAT-Cre mice (8-10 weeks, male) and CD1 mice (30-35 weeks, male) were used in this study and they were given access to food and water ad libitum. The mice were housed under a 12 h light-dark cycle, at consistent humidity (50 ± 5 %), and ambient temperature (22-25°C). The number of mice used for each experiment is listed in figure legends.
Wild animals	The study did not use wild animals.
Field-collected samples	The study did not involve samples collected from the field.
Ethics oversight	All procedures were approved by the Animal Care Committee at Huazhong University of Science and Technology and performed in accordance with the Institutional Animal Welfare Guidelines.

Note that full information on the approval of the study protocol must also be provided in the manuscript.
